# Unusual MRI abnormality in patients with nonketotic hyperglycaemia-associated seizures

**DOI:** 10.1093/bjrcr/uaae034

**Published:** 2024-09-09

**Authors:** Amine Bentahar, Habib Bellamlih, Khalil Chafi, Monsif Salek, Soufiane Belabbes, Brahim Zinoun, Taoufik Africha

**Affiliations:** Radiology Department, Moulay Ismail Military Hospital, Meknes, 50000, Morocco; Department of Radiology, Sidi Mohamed Ben Abdellah University, Fez, 30000, Morocco; Radiology Department, Moulay Ismail Military Hospital, Meknes, 50000, Morocco; Department of Radiology, Sidi Mohamed Ben Abdellah University, Fez, 30000, Morocco; Radiology Department, Moulay Ismail Military Hospital, Meknes, 50000, Morocco; Department of Radiology, Sidi Mohamed Ben Abdellah University, Fez, 30000, Morocco; Radiology Department, Moulay Ismail Military Hospital, Meknes, 50000, Morocco; Department of Radiology, Sidi Mohamed Ben Abdellah University, Fez, 30000, Morocco; Radiology Department, Moulay Ismail Military Hospital, Meknes, 50000, Morocco; Department of Radiology, Sidi Mohamed Ben Abdellah University, Fez, 30000, Morocco; Radiology Department, Moulay Ismail Military Hospital, Meknes, 50000, Morocco; Department of Radiology, Sidi Mohamed Ben Abdellah University, Fez, 30000, Morocco; Radiology Department, Moulay Ismail Military Hospital, Meknes, 50000, Morocco; Department of Radiology, Sidi Mohamed Ben Abdellah University, Fez, 30000, Morocco

**Keywords:** magnetic resonance imaging, nonketotic hyperglycaemia, seizures, subcortical T2/FLAIR hypointensity

## Abstract

Nonketotic hyperglycaemia (NKH) is a metabolic disorder typically observed in individuals with inadequately managed or undiagnosed diabetes mellitus (DM). Seizures are a common clinical presentation in NKH, and they tend to respond better to glucose correction than anticonvulsant therapy. MRI scans may reveal both subcortical T2/fluid-attenuated inversion recovery (FLAIR) imaging hypointensity and cortical changes, including cortical grey matter T2/FLAIR imaging hyperintensity and cortical or leptomeningeal enhancement, although cortical abnormalities are less frequently observed. These alterations are reversible when the underlying metabolic disturbance is effectively addressed. We suggest the role of iron accumulation as a mechanism for subcortical T2 hypointensity using T2* weighted imaging. Our cases substantiate the significance of subcortical T2/FLAIR hypointensity as a fundamental feature of this condition. In the appropriate clinical context, the recognition of these MRI abnormalities can help prevent misdiagnosis and facilitate timely treatment.

## Clinical presentation

Case 1: A 55-year-old patient with type 2 diabetes, treated with insulin therapy, presented with respiratory symptoms and fever in the pandemic context of COVID-19. A rapid antigen polymerase chain reaction (PCR) test confirmed COVID-19 infection. The patient was treated according to the recommended protocol with antibiotics (azithromycin) and corticosteroids, along with symptomatic treatment. A good clinical and biological evolution was marked, with diabetic control difficulties due to corticosteroid therapy during his stay in hospital. After which the patient presented 2 episodes of partial epileptic seizure affecting the left side of his body, each are lasting approximately 3 min, with an hour interval between the seizures. The patient also had post-seizure paresthaesia. The patient was conscious, afebrile, and had a normal neurological examination.

Case 2: A young 23-year-old patient, with no significant medical history, particularly no known diabetes, presented at the emergency department with a series of generalized tonic-clonic seizures that recurred 3 times. Each seizure lasted approximately 1 min and resolved spontaneously. Upon clinical examination, the patient was conscious, afebrile, and displayed no neurological abnormalities.

## Investigation and imaging findings

Case 1: Brain CT scan showed no abnormalities. The laboratory tests revealed a blood glucose level of 402 mg/dl, HbA1c of 94 mmol/mol, and sodium level of 136 mEq/l, with a calculated plasma osmolarity of 311 mosm/l. Acetone was not detected in the blood. During an electroencephalogram (EEG) recording, an epileptic episode was recorded, with ictal discharges in right temporo-parieto-occipital regions. The patient later benefitted from an MRI, revealing white matter hypointensity with cortical hypersignal in the right temporo-parieto-occipital regions on T2 and FLAIR imaging. The MRI also showed hypointensity in the white matter on susceptibility-weighted imaging and cortical hypersignal on diffusion-weighted imaging in the same regions.

Case 2: The patient underwent a CT scan, which returned with no abnormalities. Laboratory tests revealed a blood glucose level of 235 mg/dl, HbA1c of 75 mmol/mol, and a sodium level of 136 mEq/l, with a calculated plasma osmolarity of 285 mosm/l. Ketones were not detected. EEG registration recorded an ictal episode characterized by spike-wave discharges in the bilateral frontoparietal region. Subsequently, the patient underwent a brain MRI, which showed a hypointense signal on T2-weighted, FLAIR imaging, and susceptibility-weighted imaging in the bilateral frontal white matter region and the genu of the corpus callosum, without a corresponding cortical hyperintensity on diffusion-weighted imaging in the same areas.

## Differential diagnosis

We investigated the presence of microhaemorrhage or posterior reversible encephalopathy syndrome (PRES) due to the hypointensity observed on T2-MRI and T2*-MRI in the temporoparietal lobe. However, the hypointensity on T2*-MRI in our case did not have a spotty appearance, and its boundary was unclear. Additionally, the patient’s blood pressure was not high, which is typical for PRES. Moreover, PRES usually manifests as T2 hyperintensity and not as subcortical T2 hypointensity. As a result, we confirmed that the diagnosis was seizure-associated with nonketotic hyperglycaemia (NKH).

## Treatment, outcome, and follow-up

Both cases received rehydration with correction of hydroelectrolytic disorders and insulin therapy according to blood glucose levels, together with anticonvulsants.

Further seizures were observed before glycaemic normalization with insulin therapy was obtained, the evolution was marked by the disappearance of seizures.

They are still being followed, and after adequate diabetic control, the 2 patients no longer had convulsive seizures. A follow-up MRI in the first case at 1 year showed total regression of the abnormalities.

## Discussion

NKH is a serious acute complication of diabetes mellitus (DM), typically in patients over 50 years with type 2 DM. Seizures are a common occurrence in patients with NKH, previous studies, and reported cases have suggested that slightly elevated serum osmolarity may play a role in inducing seizures, pointing to the significance of long-standing hyperglycaemia with abnormal HbA1c levels rather than an acute hyperglycaemic hyperosmolar state.^1^

NKH is known to be associated with a variety of neurological manifestations, such as delirium, seizures, hemichorea-hemiballism, dysphagia, hemianopia, hemiparesis, and hemisensory loss. The majority of seizures observed in NKH are classified as partial seizures, sometimes with secondary generalization, and unfortunately, they often do not respond well to antiepileptic medications, posing a treatment challenge.^2^

The hyperglycaemic non-ketotic state exerts a proconvulsant effect by reducing the epileptic threshold through increased gamma-aminobutyric acid (GABA) metabolism. Interestingly, during the hyperglycaemic state, brain activity related to the Krebs cycle and glucose utilization decreases, prompting a shift in cerebral metabolism towards alternative pathways. Unlike ketoacidosis, where ketones serve as an alternative energy source, NKH leads to the brain utilizing GABA to produce succinic acid.^1^

Seizures linked to NKH may coincide with distinctive MRI abnormalities, including subcortical T2 hypointensity in the white matter, with hypointensity sometimes seen on T2*-weighted or susceptibility weighted imaging (SWI) images, Cortical hyperintensity on T2/FLAIR images with restricted diffusion, often associated with gyral or leptomeningeal gadolinium enhancement. These MRI findings are reversible as the patient clinically recovers.^1–3^

In the MRI scans, all our patients exhibited subcortical hypointensity on T2/FLAIR sequences in a specific region corresponding to the ictal focus identified in the EEG. The first case also showed subtle hyperintensity in the overlying cortex.

The leptomeningeal enhancement is likely due to vasodilation caused by seizure-induced hypermetabolism and increased brain perfusion. Prolonged ictal activity may lead to hypoxia and acidosis, disrupting the blood-brain barrier and causing contrast medium leakage, resulting in cortical enhancement. The reason for subcortical T2 shortening on imaging remains unclear, but potential mechanisms include ischaemia, mineral deposition, free radicals, or iron accumulation due to excitotoxicity and axonal damage during seizures. Hypointense signals on SWI in corresponding areas support this hypothesis, as low SWI signal intensity can indicate haemorrhage, mineral accumulation, increased vascularity, or heightened oxygen extraction related to ischaemia. Striatal hypointensities in SWI have also been reported in hyperglycaemia-induced hemichorea-hemiballism, possibly related to non-heme iron, manganese, and zinc deposition within the gemistocytes.^4^

This uncommon feature is reported in the literature even as an isolated imaging finding without cortical involvement. The hypointense signal on T2-weighted, FLAIR imaging, and susceptibility-weighted imaging is typically unilateral and focal, predominantly affecting parieto-occipital and perirolandic regions, and shows a strong correlation with the EEG focus.^1^

The primary treatment approach involves maintaining good glycaemic control, ensuring proper hydration, and administering anti-seizure medications. However, the necessity of long-term medication use has not been definitively established. It is essential to note that phenytoin, an anti-seizure medication, can potentially exacerbate hyperglycaemia and, in some reported cases, has been ineffective in controlling seizures.^3^

## Conclusion

NKH is a metabolic disorder typically observed in individuals with inadequately managed or undiagnosed DM. Seizures often manifest as a common clinical symptom in NKH, and they tend to respond better to glucose correction than anticonvulsant therapy. MRI scans may reveal both subcortical T2/FLAIR hypointensity and cortical changes.

## Learning points

- Nonketotic hyperglycaemia (NKH) is known to cause focal motor seizures with or without secondary generalization.
- MRI can reveal subcortical T2/FLAIR hypointensity and cortical alterations, such as grey matter T2/FLAIR hyperintensity and cortical or leptomeningeal enhancement. It’s important to note that cortical abnormalities are less frequently observed.
- In the right clinical context; for instance: in a patient with seizures with high blood sugar levels, elevated HbA1c, and no ketoacidosis, identifying these MRI abnormalities can help prevent misdiagnosis and facilitate timely treatment.
